# TLR7 Is Critical for Anti-Viral Humoral Immunity to EV71 Infection in the Spinal Cord

**DOI:** 10.3389/fimmu.2020.614743

**Published:** 2021-02-18

**Authors:** Ya-Lin Lin, Mei-Yi Lu, Chi-Fen Chuang, Yali Kuo, Hong-En Lin, Fu-An Li, Jen-Ren Wang, Yi-Ping Hsueh, Fang Liao

**Affiliations:** ^1^ Institute of Biomedical Sciences, Academia Sinica, Taipei, Taiwan; ^2^ National Institute of Infectious Diseases and Vaccinology, National Health Research Institutes, Tainan, Taiwan; ^3^ Department of Medical Laboratory Science and Biotechnology, National Cheng Kung University, Tainan, Taiwan; ^4^ Department of Pathology, National Cheng Kung University Hospital, Tainan, Taiwan; ^5^ Center of Infectious Disease and Signaling Research, National Cheng Kung University, Tainan, Taiwan; ^6^ Institute of Molecular Biology, Academia Sinica, Taipei, Taiwan

**Keywords:** TLR7, EV71, humoral immunity, spinal cord, antibody

## Abstract

Enterovirus 71 (EV71) is a positive single-stranded RNA (ssRNA) virus from the enterovirus genus of *Picornaviridae* family and causes diseases ranged from the mild disease of hand, foot and mouth disease (HFMD) to the severe disease of neurological involvement in young children. TLR7 is an intracellular pattern recognition receptor (PRR) recognizing viral ssRNA. In this study, we investigated the role of TLR7 in EV71 infection in mouse pups (10-12 days old) and found that wild-type (WT) and TLR7 knock-out (TLR7KO) mice infected with EV71 showed similar limb paralysis at the onset and peak of the disease, comparable loss of motor neurons, and similar levels of antiviral molecules in the spinal cord. These results suggest that TLR7 is not the absolute PRR for EV71 in the spinal cord. Interestingly, TLR7KO mice infected with EV71 exhibited significantly delayed recovery from limb paralysis compared with WT mice. TLR7KO mice infected with EV71 showed significantly decreased levels of IgM and IgG2, important antibodies for antiviral humoral immunity. Furthermore, TLR7KO mice infected with EV71 showed a decrease of germinal center B cells in the spleen compared with WT mice. Altogether, our study suggests that TLR7 plays a critical role in anti-viral humoral immunity rather than in being a PRR in the spinal cord during EV71 infection in young mice.

## Introduction

Enterovirus 71 (EV71) is a positive single-stranded RNA (ssRNA) virus from the enterovirus genus of *Picornaviridae* family (1) and causes hand, foot and mouth disease (HFMD) in young children (2). The illness caused by EV71 infection can range from mild symptom with fever and erythrasma to severe symptom with neurological involvement and life-threatening complication (3, 4). The neurological manifestations range from meningitis to acute flaccid paralysis and brainstem encephalitis, leading to systemic disorders including severe pulmonary edema and heart failure (5). Moreover, some children recovering from severe symptoms have been reported to develop neurological sequelae (6). However, the neurological pathogenesis caused by EV71 infection remains poorly understood.

Infants and young children are most susceptible and vulnerable to EV71 infection. Notably, neonatal immune responses are thought immature and quite different from those of adults. Because the adaptive immunity is not fully mature in neonatal, the innate immunity to EV71 infection becomes very important for those infants as well as young children. Hosts recognizing pathogens through pattern recognition receptors (PRRs) initiate innate immune responses to specific pathogens as the first line of host defense followed by dictating adaptive immunity to pathogens for eliminating pathogens (7–9). PRRs include Toll like receptors (TLRs), RIG-I like receptors (RLRs) and C-type lectin like receptors (CLRs) (10). Among PRRs, the endosomal TLRs including TLR3, TLR7/8 and TLR9, and cytosolic RLRs including retinoic acid-inducible gene I (RIG-I) and melanoma differentiation-associated gene 5 (MDA5) play important roles in sensing viral RNA during viral infection (7, 9–11). The selective endosomal PRRs and RLRs during virus infection are various from virus to virus dependent on virus’ nucleic acid structure. TLR3 recognizes double-stranded RNA viruses (12–14), whereas TLR7 and TLR8 recognize ssRNA viruses (15, 16). RLRs including RIG-I and MDA5 are cytosolic helicases and detect dsRNA intermediates accumulating in the cytosol during viral replication (17). The *in vivo* study on the PRRs employed by EV71 infection has been rarely studied (18). Given that EV71 is a positive ssRNA virus, TLR7 and TLR8 as well as RLRs can be the potential candidate since these PRRs recognize ssRNA virus. Performing EV71 infection in mice deficient in molecules downstream of PRR signaling revealed that MyD88- and IRF7-knockout mice, but not mitochondrial anti-viral signaling protein (MAVS)-knockout mice, were susceptible to EV71 infection (unpublished data), making TLR7 and TLR8 potential candidate PRRs for EV71. Murine TLR8 was initially thought to be non-functional because mouse TLR8 did not respond to TLR8 ligand (19). However, murine TLR8 later has been demonstrated functional (20). Given that controversial reports on TLR8 function in mice (19, 20), we decided to study the effect of TLR7 on EV71 infection using TLR7-deficient mice. In this study, we have demonstrated that EV71 mainly targets motor neurons in the spinal cord, that TLR7 is not the absolute PRR for EV71 infection in the spinal cord, and that TLR7KO mouse pups show significantly delayed recovery from EV71-induced limb paralysis without significant difference at the onset and peak of disease compared with WT mice. We have further demonstrated that TLR7KO mice show significantly reduced levels of IgG2a/c and IgG2b, important IgG subtype for anti-viral effect, in the spinal cord along with the reduced frequency and number of germinal center B cells (GC B) in the spleen during the recovery phase. The reduced GC B cells may impair humoral immunity leading to the reduced antibody production and contribute, in part, to the delayed recovery from EV71-induced limb paralysis in TLR7KO mice.

## Material and Methods

### Mice

TLR7 knockout (KO) mice (21) were purchased from The Jackson Laboratory and backcrossed with C57BL/6 mice. All mice were housed in specific pathogen free conditions at the Institute of Biomedical Sciences, Academia Sinica (Taipei, Taiwan). All animal experiments were approved by the Institutional Animal Care and Utilization Committee at Academia Sinica (IACUC protocol # 11-12-264) and performed in accordance with institutional guidelines.

### Virus Infection

Mouse pups (10-12 days old) were intraperitoneally (i.p.) injected with 8 × 10^5^ PFU per mouse of mouse-adapted EV71 (22). After receiving virus, mice were monitored daily for limb paralysis and scored with a score scale from 0 to 5: score 0, no disease; score 1, one or two hindlimb weakness (wobbling); score 2, one hindlimb paralysis; score 3, both hindlimb paralysis; score 4, both forelimb plus both hindlimb paralysis; score 5, death.

### Virus Neutralization Test

Sera from EV71-infected mice were collected and heat-inactivated at 56°C for 30 minutes. Sera were serially diluted in D2 (high glucose Dulbecco’s modified Eagle’s medium containing 2% heat-inactivated fetal bovine serum) followed by mixing with EV71 (5000 TCID) at a volume of 1:1. The serum-virus mixtures were incubated at 37°C for 2 hours followed by adding to RD cells (a human rhabdomyosarcoma cell line, ATCC, CCL-136). After 2-day culture, cells were fixed with 4% paraformaldehyde and stained with crystal violet solution. The neutralization titer was referred as the dilution titer that completely prevented cells from virus-induced cytopathic effect.

### Plaque Assay

RD cells (5 × 10^5^ cells) were seeded in each well of a 6-well plate one day before infection. Spinal cord lysates were serially diluted ten-fold in D2. Cells were washed once with Dulbecco’s PBS (DPBS) containing Ca^2+^/Mg^2+^ followed by incubation with serially diluted lysates at 37°C for 1 hour. After removal of lysates, cells were washed once with DPBS and a mixture of D2 and low melting agarose (1:1) was added to cells. Cells were incubated at 37°C for 2 days followed by fixation with 4% paraformaldehyde. After removal of agarose, cells were stained with crystal violet solution and plaques in each well were counted to determine the virus titer.

### Gene Expression Analysis by Quantitative PCR

Total RNA of the spinal cord was extracted using Trizol reagent (Invitrogen) according to the manufacturer’s instructions. Total RNA was treated with RQ1 RNase-free DNase (Promega), and the first strand of cDNA was synthesized by using random hexamers and SuperScript III reverse Transcriptase (Invitrogen). Quantitative PCR was performed on ABI prism 7500 (Applied Biosystems) using SYBR Green Master mix (Thermo Scientific). The thermal cycling protocol was 1 cycle at 50°C for 2 minutes and 95 °C for 10 minutes, followed by 45 cycles of 95°C for 15 seconds and 60 °C for 1 minute. Gene expression was normalized to GAPDH and analyzed using the ΔCt method. DNA sequences of primer pairs used in this study are listed in the **Supplementary Table 1**.

### Immunofluorescence Staining

Mice were anesthetized by the inhalation of isoflurane vapor. A proper anesthesia is confirmed by the lack of a deep tendon reflex. The mouse blood was collected from heart and then perfused transcardially with PBS and 4% paraformaldehyde. The spinal cord was immediately dissected and post-fixed with 4% paraformaldehyde in PBS at 4°C for 24 hours followed by incubation in 30% sucrose/PBS at 4°C overnight. The spinal cords were embedded in OCT (Sakura Finetek) and 16 µm cross-sections were obtained using a cryostat (Leica CM3050). Before immunostaining, floating frozen sections were immersed in 30% sucrose/PBS for 1 hour, then placed onto gelatin-coated slide and dried overnight. Spinal cord sections were performed antigen retrieval using DAKO retrieval solution S1700, in which sections were boiled for 20 minutes in a pressure pot and then cooled down for another 20 minutes. Spinal cord sections were washed for 30 minutes in PBS and blocked at room temperature for 1 hour in blocking solution (1% BSA, 0.3% Triton X-100 in PBS). Spinal cord sections were then incubated with primary antibodies in blocking solution at 4°C overnight. After PBS washing, the sections were incubated with secondary antibodies at room temperature for 1 hour. After washing for 30 minutes in PBS, spinal cord sections were counterstained with DAPI, mounted and observed under confocal microscopy (LSM800 with Airyscan, ZEISS). Antibodies used in this study are listed in the **Supplementary Table 2**.

### ELISA

Immunoglobulin concentrations in spinal cord lysates and sera were determined by sandwich ELISA assay. In short, ELISA plates were coated with 2 µg/ml of goat anti-mouse Ig (SouthernBiotech) in 50 mM carbonate/bicarbonate buffer. Plates were washed with PBST (PBS containing 0.05% Tween 20) followed by blocking with 0.5% BSA in PBS at room temperature for at least 2 hours. The spinal cord lysates and sera were serially diluted and added to the plates followed by incubation at 4°C overnight. Mouse IgG1 (BioLegend), IgG2b (BioLegend), IgG2c (GeneTex), IgG3 (BioLegend) and IgM (Biolegend) were also serially diluted and added to the plates as standards. After several washes with PBST, plates were incubated with HRP-conjugated goat anti-mouse IgG1, IgG2b, IgG2c, IgG3 or IgM (SouthernBiotech) at room temperature for 1 hour. Plates were then washed several times with PBST followed by incubation with ELISA substrate solution 3,3′,5,5′-tetramethylbenzidine substrate (KPL). The reaction was stopped by 1M H_2_SO_4_ and the optical density (OD) was measure at 450 nm on SpectraMax 190 (Molecular Devices).

### Flow Cytometry Analysis

Mice were sacrificed and perfused with PBS followed by isolation of spinal cords. Spinal cords were dissociated with enzymatic or mechanical methods depending on the desired cell population for FACS analysis. For the analysis of neurons, astrocytes and oligodendrocytes, spinal cords were enzymatically dissociated using Neural tissue dissociation kit-postnatal neurons (Miltenyi Biotec) according to manufacturer’s instructions. For the analysis of microglia and B cells, spinal cords were mechanically dissociated in C tube by running through Program m_brain_01 to m_brain_03 on a gentle MACS Dissociator (Miltenyi Biotec). Dissociated tissues were filtered through 70 µm mesh and resuspended in medium (DMEM containing 10% FBS) containing 20% Percoll (GE Healthcare) followed by centrifugation to remove the myelin debris. The supernatant containing the myelin was discarded and the pelleted cells were washed, resuspended, and subjected to immunofluorescence staining followed by FACS analysis. The isolation of splenocytes was performed by mechanically dissociation of spleens in C tube and ran the Program m_spleen_01 on a gentle MACS Dissociator. The splenocytes were then subjected to the removal of red blood cells using lysing buffer (BD Pharm Lyse) according to manufacturer’s instructions. For cell surface staining, cells were washed with FACS buffer once (HBSS with Ca^2+^/Mg^2+^, 1% FBS, 10 mM HEPES and 0.1% NaN_3_) and blocked with Fc blocker (clone 2.4G2, BD Biosciences) on ice for 5-10 minutes. Mixtures of antibodies against cell surface markers were added to cells and incubated on ice for 30 minutes. After incubation, cells were washed twice with HBSS and stained with viability dye (eBioscience) on ice for 10 minutes followed by consecutive washes with HBSS and FACS buffer. Finally, cells were fixed in FACS buffer containing 0.8% paraformaldehyde and subjected to FACS analysis. For cell surface staining of FcγRs, cells were stained with fluorochrome-conjugated anti-mouse CD16/32, anti-mouse CD64, anti-mouse CD32b and anti-mouse CD16.2 prior to incubation with antibodies against cell surface markers. For intracellular staining of TLR7, cells were stained with antibodies to surface markers and viability dye followed by fixation in Cytofix/Cytoperm solution (BD Biosciences), and then subjected to intracellular staining using Fixation/Permeabilization solution kit (BD Biosciences) according to manufacturer’s instructions. For intracellular staining of nuclear antigen NeuN, cells were stained with antibodies to surface markers and viability dye followed by fixation in TF Fix/Perm Buffer (BD Biosciences), and then subjected to intracellular staining using Transcription Factor Buffer Set (BD Biosciences) according to manufacturer’s instructions. Flow cytometry analysis was performed on a BD LSRII and the data were analyzed using FlowJo software (BD Biosciences). Antibodies used in the study are listed in the **Supplementary Table 3**.

### Proteomic Analysis

Six samples of spinal cord lysates (100 µg each) collected from WT and TLR7KO mice at day 3, day 5 post-infection and recovery phase (recovery to score = 1) were dissolved in 6M urea, reduced with 5 mM dithiothreitol at 56°C for 25 minutes, and alkylated with 15 mM iodoacetamide at 25°C for 45 minutes. Subsequently Trypsin/Lys-C Mix (Promega, USA) was added to protein samples at a ratio of 20:1 (protein/protease, w/w), and incubated at 37°C for 4 hours. Protein samples were then diluted to a final concentration of 1 M urea with 50 mM triethylammonium bicarbonate, and continued incubating at 37°C for 17 hours. Digested samples were acidified with formic acid to a pH ∼2, desalted with C18 Oasis^®^ PRiME HLB cartridges (Waters, USA), and subjected to tandem mass tag (TMT)-labeling using TMT6-plexTM reagents (Thermo Scientific, USA). TMT reagents were dissolved in 41 µL of anhydrous acetonitrile and added to 100 µg of peptides dissolved in 100 µL of 50 mM triethylammonium bicarbonate. After one-hour incubation, the reaction was quenched by adding 8 µL of 5% hydroxylamine and incubated for 15 minutes at 37°C. Labeled peptides from six samples were combined and desalted with C18 Oasis^®^ PRiME HLB cartridges. The combined TMT-labeled peptides were solubilized in buffer A (20 mM ammonium formate, pH 10) and separated on an XBridge BEH130 C18 column (3.5 µm, 2.1 × 150 mm, Waters, USA) using a 1100 series HPLC equipped with a UV detector (Agilent, USA). The separation was performed with a 90-min gradient, 5 minutes at 5% buffer B (20 mM ammonium formate with 80% acetonitrile, pH 10), linear increase from 5 to 43% B over 45 min, followed by a linear increase to 100% B over 20 minutes, then isocratic at 100% for 5 minutes before a re-equilibration at 5% B for 15 minutes. A total of 80 × 1 minute fractions (200 µL each) were collected. The 15 pooled fractions were lyophilized before subjected to LC-MS/MS analysis. The TMT-labeled peptides were dissolved in 0.1% aqueous formic acid solution and relatively quantified by LC-MS/MS on an Easy-nLC 1200 system (Thermo Scientific, USA) coupled with an Orbitrap Fusion™ Lumos™ Tribrid™ Mass Spectrometer (Thermo Scientific, USA). Peptide mixtures were separated on an Acclaim PepMap^®^ RSLC (25 cm × 75 µm i.d., Thermo Scientific, USA) at a flow rate of 300 nL/min using a 130 min-gradient from 5% to 45% solvent B (solvent B was 0.1% formic acid in acetonitrile; Solvent A was 0.1% formic acid in water). The mass spectrometer was operated in the data-dependent mode. Full MS (m/z 350–1600) resolutions were set to 120,000 and MS2 scans acquired using the following settings: quadrupole isolation window 0.7; CID MS2 fragmentation and detection in the ion trap with AGC target at 1.0 × 104, collision energy set at 30%; HCD MS2 detection in the orbitrap with AGC target at 5.0 × 104, collision energy set at 35%, resolutions were set to 50,000 FWHM at m/z 200). The raw files were processed with Proteome Discoverer v.2.2 (Thermo Scientific, USA) for protein identification and TMT-based relative protein quantification. Database search through the Mascot search engine v.2.6.1 (Matrix Science) against the mouse proteome FASTA files (2019-05, 62,656 entries) downloaded from UniProt database. The search was performed according to the following parameters: precursor and fragment mass tolerances of 10 ppm and 0.02 Da, 2 missed cleavage sites, cysteine carbamidomethylation and TMT6plex (lysine, peptide N-terminus) as fixed modifications, variable oxidation of methionine, variable deamidation of asparagine or glutamine. The peptide spectrum matches are verified by q-values (1% false discovery rate) from the Percolator algorithm in the Proteome Discoverer based on a decoy database search. Protein quantification was accomplished by assessing the relative signal-to-noise values of reporter ions extracted from MS2 spectra. The dataset presented in this study can be found in MassIVE with accession number: MSV000086240.

### Statistical Analysis

The Mann-Whitney U test was used to compare the differences between two groups. The Log-rank test was used for the analysis of survival rates. *P-*values < 0.05 were considered statistically significant. Statistical analysis was performed using GraphPad Prism software version 8.

## Results

### TLR7KO Mouse Pups Infected With EV71 Show Significantly Delayed Recovery From Limb Paralysis Compared With WT Mice

**Figure 1:**
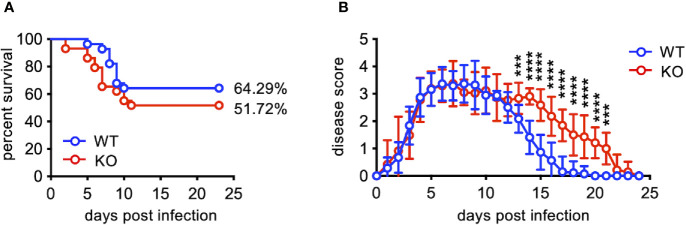
TLR7KO mice show delayed recovery from EV71-induced limb paralysis. Mouse pups (10-12 days old) of WT (n=23) and TLR7KO (n=29) were infected with EV71 virus (8 ×10^5^ PFU) and monitored daily for clinical signs of limb paralysis. **(A)** Survival analysis of EV71-infected WT and TLR7 KO mice. The Log-rank test, *p* = 0.2392. **(B)** The clinical scores of EV71-infected WT and TLR7KO mice are shown. Score 0, no disease; score 1, one or two hindlimb weakness (wobbling); score 2, one hindlimb paralysis; score 3, both hindlimb paralysis; score 4, both forelimb plus both hindlimb paralysis; score 5, death. Data shown are from four independent experiments. Data are shown as mean ± SD. ***p < 0.001, ****p < 0.0001 by Mann-Whitney U test.

EV71 infection causing a neurological involvement is a severe threat for infants and young children (2, 3). Like humans, adult mice are resistant to EV71 infection, and only neonatal mice are susceptible to EV71 infection. To study the effect of TLR7 deficiency on EV71 infection, we infected WT and TLR7KO mouse pups (10-12 days old) with EV71 (8 × 10^5^ PFU) and assessed the survival rate and clinical scores. TLR7KO mice tended to have lower survival rate compared with WT mice (51.72% vs. 64.29%) (**Figure 1A**). Interestingly, TLR7KO mice showed comparable disease onset (day 3 post-infection) and peak disease (day 6-7 post-infection), but significantly delayed recovery from limb paralysis (**Figure 1B**).

### EV71 Infects Mainly Motor Neurons in the Ventral Horn of Spinal Cords, Resulting in the Loss of Motor Neurons in WT and TLR7KO Mouse Pups

**Figure 2:**
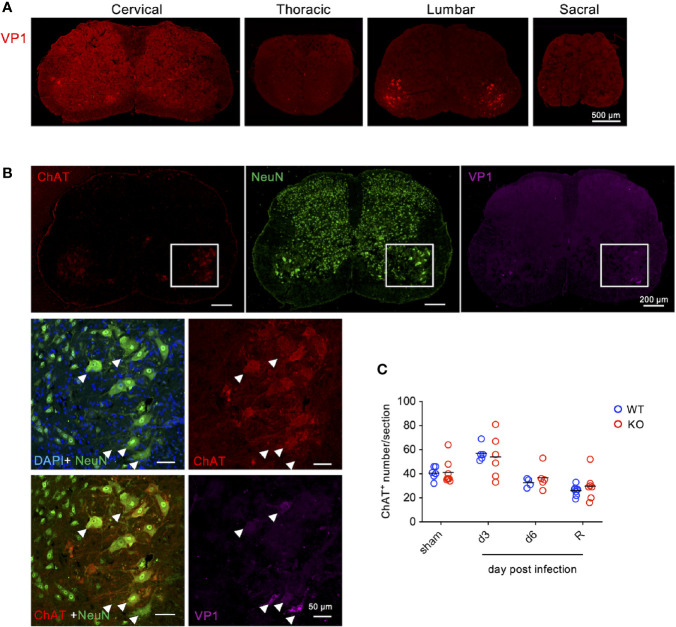
EV71 mainly infects motor neurons in the ventral horn of spinal cords. Cryosections of the spinal cord from EV71-infected mice (day 3 post-infection) were subjected to immunofluorescence staining with antibodies to viral protein VP1 along with antibodies to ChAT (motor neuron marker) and NeuN (neuron marker). **(A)** VP1 was mainly detected in the ventral horn of the lumbar spinal cord. **(B)** VP1-positive cells in the spinal cord were also positive for ChAT and NeuN. The arrow-head indicates the virus-infected motor neurons. **(C)** Total numbers of ventral horn motor neurons (ChAT-positive with large cell body) of the lumbar spinal cord from each section were counted. Each symbol represents the cell number from each section (sections were collected from at least 3 mice). R represents mice that had been recovered from limb paralysis and wobbled (score 1).

Given that EV71 is considered as a neurotropic enterovirus causing neural involvement in severe cases (3, 4) as well as in mouse models (23–25) and that TLR7KO mice show significantly delayed recovery from limb paralysis (**Figure 1**), we then investigated whether TLR7KO mice infected with EV71 showed more severe infection in neurons compared with WT mice. Staining tissue sections of the spinal cord with antibody to viral protein (VP1) of EV71, we found that VP1 was detected in the ventral horn of cervical, thoracic and lumbar spinal cords, and among them, lumbar spinal cords were more severely infected (**Figure 2A** and **Supplementary Figure 1A**). Further examining the type of cells susceptible to EV71 infection, we found that VP1-positive cells characterized by a large cell body were co-stained with choline acetyltransferase (ChAT^+^), a marker for motor neurons, indicating that EV71 infected mainly motor neurons in the ventral horn of lumbar spinal cords (**Figure 2B**). These results indicate that EV71 targets ventral horn motor neurons (lower motor neurons) in the spinal cord. Since paralysis is a typical clinical symptom of lower motor neuron lesions, EV71 infecting lower motor neurons may explain the paralysis of limbs in EV71-infected mouse pups (**Figure 1**). However, of interest, EV71-induced loss of lower motor neurons in TLR7KO and WT mice was comparable (**Figure 2C**) at the onset of disease, the peak of disease and even the recovery phase (score =1).

### TLR7 Is Dispensable as a PRR for EV71 Infection in the Spinal Cord

**Figure 3:**
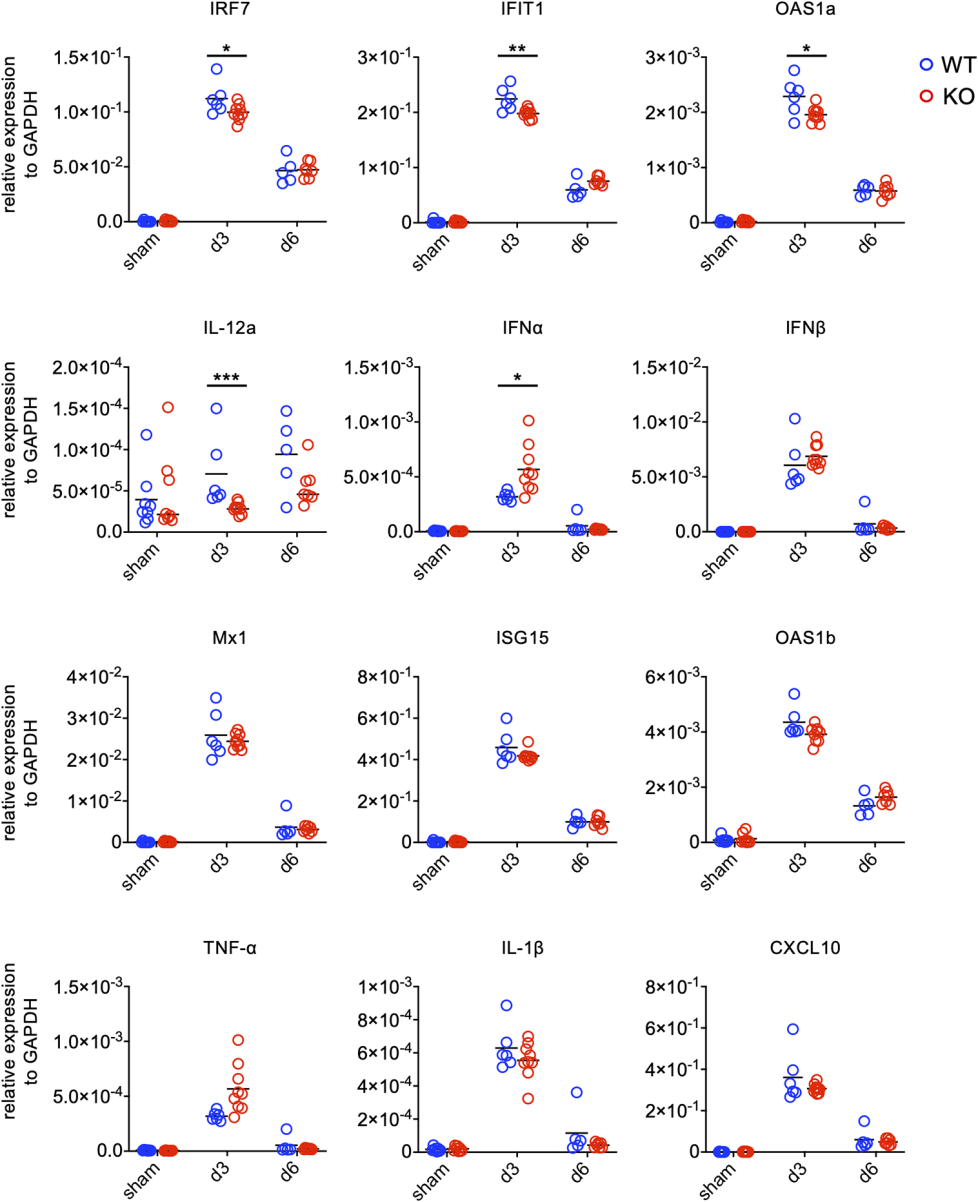
Gene expression of antiviral molecules and inflammatory cytokines in the spinal cord of EV71-infected mice. Mice were sacrificed at day 3 and day 6 post-infection. Spinal cords were collected and subjected to total RNA isolation followed by first-strand cDNA synthesis. The levels of gene expression were analyzed by real-time quantitative PCR and normalized to GAPDH. Each symbol represents one mouse. *p < 0.05, **p < 0.01, ***p < 0.001 by Mann-Whitney U test.

Given that TLR7 has been shown as a PRR for various ssRNA viruses (15, 16) and that EV71 is a ssRNA virus, we examined whether TLR7 is a PRR for EV71 infection in the spinal cord using TLR7KO mice. We analyzed the expression levels of anti-viral molecules associated with TLR7 downstream signaling as well as proinflammatory cytokines in the spinal cord by quantitative PCR at day 3 and 6 post-infection. At day 3 post-infection, both WT and TLR7KO mice showed the induction of genes associated with anti-viral effects and genes associated with proinflammatory cytokines and chemokines. The levels of genes associated with anti-viral effects were significantly reduced (*IRF7*, *IFIT1*, *OAS1a*, *IL-12a*), while some anti-viral genes (*Mx1*, *ISG15*, *OAS1b*) were somewhat reduced without reaching statistical difference in the spinal cord of TLR7KO mice compared with WT mice at day 3 post-infection (**Figure 3**). Similarly, EV71 infection also significantly induced some proinflammatory cytokines and chemokines in both WT and TLR7KO mice. However, proinflammatory cytokines (*TNF-α*, *IL-1β*) and chemokine (*CXCL10*) were somewhat lower levels in TLR7KO mice than WT mice at day 3 post-infection, but did not reach statistical significance. Strangely, TLR7KO mice showed increased expression of *IFN-α* and *IFN-β* despite the significant decrease of IRF7, a transcription factors important for type I IFN production. The levels of anti-viral molecules, cytokines and chemokines significantly waned at day 6 post-infection in both WT and TLR7KO mice. Furthermore, the induction of anti-viral molecules downstream of TLR7 signaling by EV71 in WT and TLR7KO mice did not seem to have significant impact on viral clearance since the viral titer is comparable (**Supplementary Figures 1B, C**), suggesting that TLR7 may not be the absolute PRR for recognizing EV71 in the motor neurons.

### TLR7 Expressing Cells in the Spinal Cord Are Microglia, But Not Astrocytes, Oligodendrocytes and Motor Neurons

**Figure 4:**
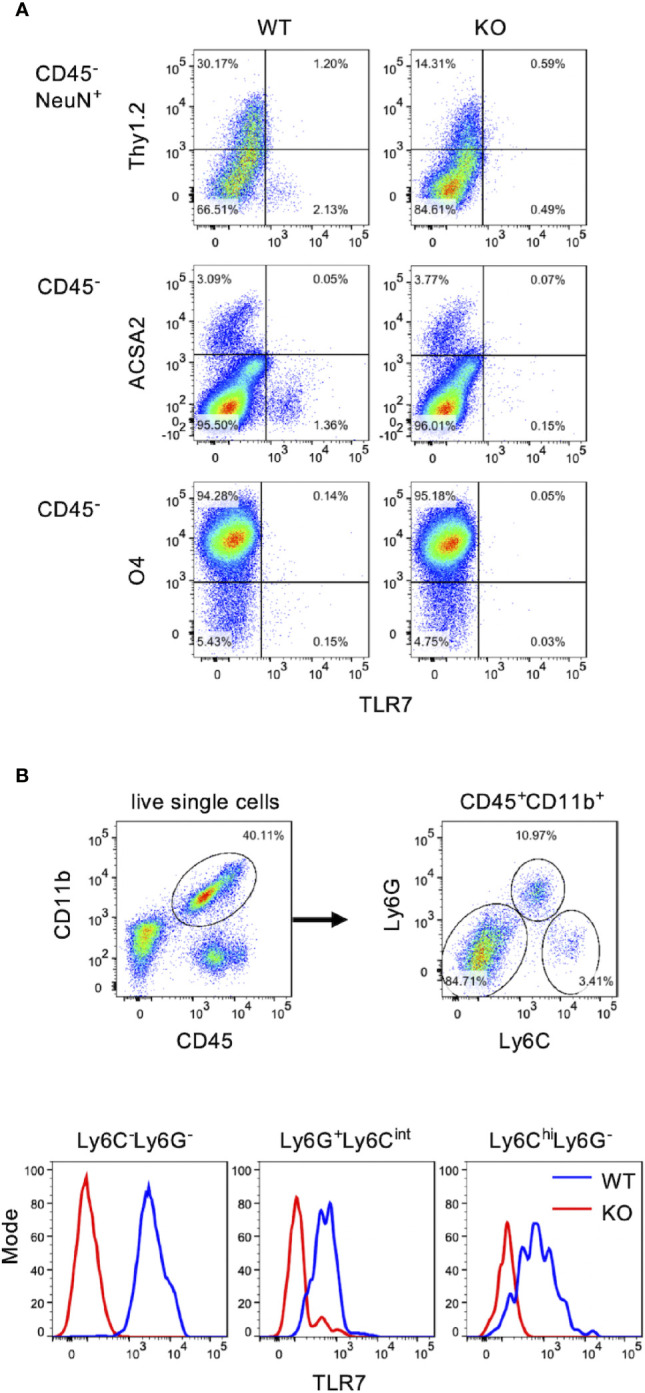
Microglia are the major TLR7-expressing cells in the spinal cord. Total cells of the spinal cord from uninfected WT and TLR7KO mice were harvested and subjected to cell surface staining with antibodies to CD45, Thy1.2, CD11b, Ly6C, Ly6G, ACSA2 (astrocyte marker) and O4 (oligodendrocyte marker) followed by intracellular staining with antibodies to TLR7 and NeuN (neuron marker), and analyzed by FACS. **(A)** Motor neurons were identified as CD45^-^NeuN^+^Thy1.2^+^ cells (motor neurons). Astrocytes were identified as CD45^-^ACSA2^+^ cells and oligodendrocytes were identified as CD45^-^O4^+^ cells. **(B)** Myeloid cells were identified as CD45^+^CD11b^+^ cells and were further divided into three populations: Ly6C^-^Ly6G^-^ (microglia), Ly6G^+^Ly6C^int^ (granulocytes) and Ly6C^hi^Ly6G^-^ (monocytes).

Examining the spinal cord cells expressing TLR7, we performed FACS analysis by staining spinal cord cells with antibody against TLR7 to identify TLR7 expressing cells. Motor neurons marked with NeuN^+^Thy1.2^+^ (26, 27), astrocytes marked with ACSA2, and oligodendrocytes marked with O4 were all TLR7 negative (**Figure 4A**). In contrast, TLR7 was highly expressed in microglia (CD45^+^CD11b^+^Ly6C^-^Ly6G^-^) (**Figure 4B**, lower left panel). The facts that EV71 targets motor neurons (**Figure 2**) and motor neurons do not express TLR7 (**Figure 4A**) may explain why TLR7 is not the PRR for EV71-infected motor neurons. Thus, TLR7 may play a role in microglia instead of motor neurons during EV71 infection in the spinal cord. We also noticed that granulocytes (Ly6G^+^Ly6C^int^) and monocytes (Ly6C^hi^Ly6G^-^), which were only small percentage of cells in the spinal cord, were also positive for TLR7 (**Figure 4B**).

### TLR7KO Mice Show Decreased Concentrations of IgM and IgG in Sera as Well as in the Spinal Cord During EV71 Infection

**Figure 5:**
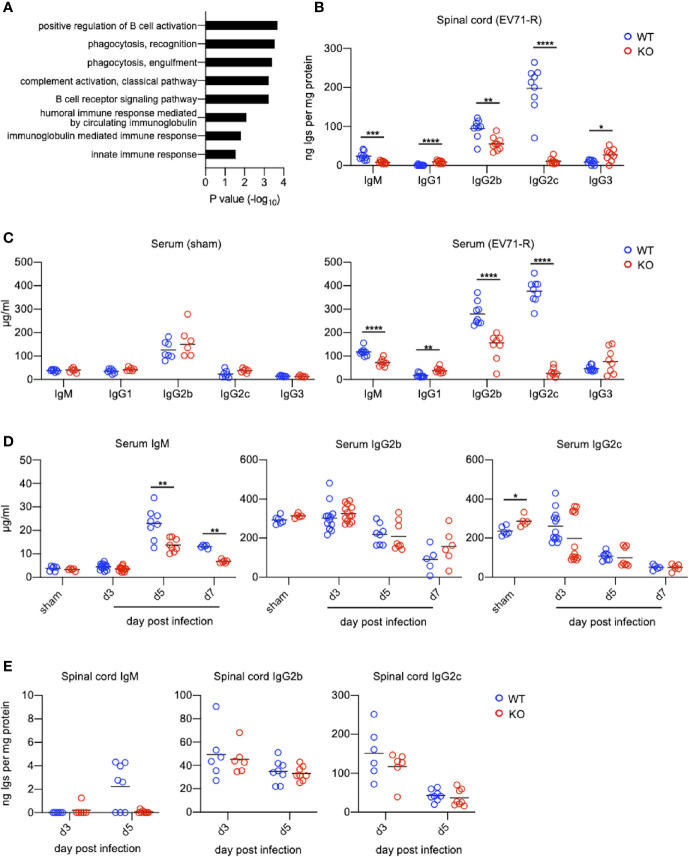
TLR7KO mice show reduced levels of IgG2b, IgG2c and IgM in spinal cords and sera during EV71 infection. **(A)** Spinal cord lysates of EV71-infected mice were subjected to TMT-labeling followed by LC/MS/MS analysis. Proteins with abundance ratio (KO/WT) <0.75 fold during recovery phase were selected and subjected to Gene Ontology (GO) analysis using DAVID v6.8. Gene ontologies representing biological process are shown. P value was tested with a modified Fisher’s Exact test. **(B**, **C)** Spinal cord lysates and sera of EV71-infected mice during recovery phase (score = 1) at day 12 to 15 (WT) or day 12 to 19 (KO) post-infection were subjected to ELISA for analyzing immunoglobulin concentrations. Immunoglobulin concentrations in the spinal cord lysates are shown **(B)**. Immunoglobulin concentrations in sera from sham-infected WT and TLR7KO mice (28-day-old mice equivalent to the mouse age of day 16 post-infection) (**C**, left panel), and EV71-infected WT and TLR7KO mice during recovery phase (EV71-R) (**C**, right panel) are shown. **(D**, **E)** Concentrations of IgM, IgG2b and IgG2c in mouse sera **(D)** and spinal cord lysates **(E)** during disease are shown. Each symbol represents one mouse. *p < 0.05, **p < 0.01, ***p < 0.001, ****p < 0.0001 by Mann-Whitney U test.

The most significant difference on EV71 infection between WT mice and TLR7KO mice is that TLR7KO mice showed significantly delayed recovery from the limb paralysis. To search for molecules responsible for the recovery from limb paralysis and affected by TLR7 deficiency, we employed the proteomic techniques to analyze molecules of spinal cords from EV71-infected WT and TLR7KO mice during the recovery phase (mice recovered to the clinical score 1). Proteins whose abundance of the ratio of KO/WT < 0.75 (**Supplementary Table 4**) were subjected to Gene Ontology (GO) analysis. Interestingly, the GO result revealed that proteins, which significantly reduced in the spinal cord of EV71-infected TLR7KO mice as compared with WT mice, were mainly involved in the biological process on phagocytosis and humoral immune responses (**Figure 5A**). Proteomic data showed that antibodies (IgM and IgG) were significantly reduced in the spinal cord of TLR7KO mice compared with WT mice (**Supplementary Table 4**). Performing ELISA analysis, we found that TLR7KO mice infected with EV71 significantly reduced IgM levels in the spinal cord compared with WT mice during the recovery phase (**Figure 5B**). Interestingly, TLR7KO mice exhibited a decrease of IgG2b and IgG2c while an increase of IgG1 and IgG3 in the spinal cord as compared with WT mice (**Figure 5B**). Similarly, we also found that TLR7KO mice infected with EV71 showed an increase of serum IgM, IgG2b and IgG2c while a decrease of IgG1 and IgG3 compared with EV71-infected WT mice during the recovery phase (**Figure 5C**). Of note, we noticed that the high concentrations of IgG isotypes were present at day 3 and day 5 post-infection (equivalent to day 14 and day 16 postnatal development) in both TLR7KO and WT mice (**Figures 5D, E**). The levels of antibodies were comparable in WT and TLR7KO mice at day 3, day 5 and day 7 post-infection (**Figures 5D, E**). As expected, the serum IgG2b and IgG2c gradually declined as time went on (**Figure 5D**), supporting that they were presumably maternal antibodies. Of note, a mature pattern of immune response to antigens will not be achieved until 3-4 weeks postnatal development (28, 29). Nevertheless, the levels of IgM were much lower than those of IgGs at day 3 post-infection (**Figures 5D, E**). The high levels of IgGs of mouse pups were maternal IgGs, which can be transported across the placenta, while IgM were produced by mouse pups. The levels of IgM were significantly increased at day 5 and day 7 post-infection in both EV-71 infected WT and TLR7KO mice; however, the levels of IgM were significantly reduced in EV71-infected TLR7KO mice as compared with EV71-infected WT mice. These results may imply that TLR7 plays a role in humoral immunity to EV71.

### TLR7KO Mice Infected With EV71 Significantly Reduce the Number of B Cells in the Spinal Cord and Class-Switched Germinal Center B Cells in the Spleen Compared With WT Mice During EV71 Infection

**Figure 6:**
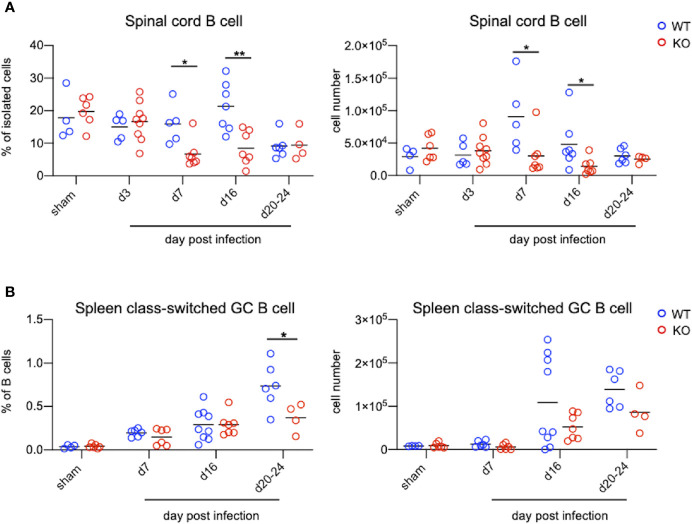
TLR7KO mice show reduced B cells in the spinal cord and reduced class-switched germinal center B cells in the spleen during EV71 infection. Spinal cord cells and splenocytes from EV71-infected mice were harvested, isolated and subjected to FACS analysis of surface staining to identify B cells. **(A)** Spinal cord B cells were identified as CD45^+^B220^+^CD11b^-^ cells. The frequency (**A**, left panel) and the number (**A**, right panel) of B cells in the spinal cord of EV71-infected mice are shown. **(B)** Spleen B cells were gated on CD45^+^B220^+^ cells. Class-switched germinal center B cells were further gated on IgD^-^IgM^-^CD95^+^CD38^int^ cells. The frequency (**B**, left panel) and cell number (**B**, right panel) of class-switched germinal center B cells in spleens of EV71-infected mice at days post-infection are shown. Each symbol represents one mouse and data shown are from 5-6 independent experiments. *p < 0.05, **p < 0.01 by Mann-Whitney U test.

**Figure 7:**
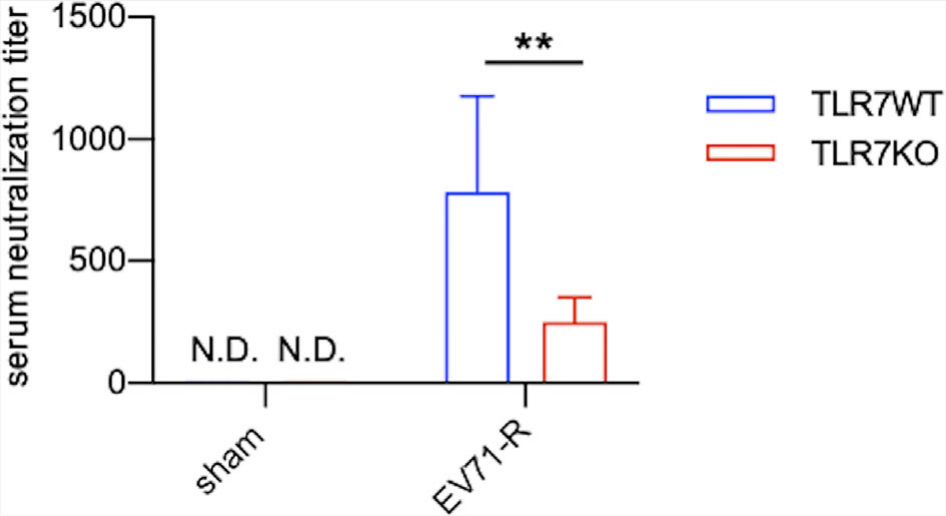
Antibodies produced by TLR7KO mice show reduced ability to neutralize EV71 virus. Sera from sham-infected WT (n=7) and TLR7KO (n=6) (28-day-old mice equivalent to the mouse age of day 16 post-infection) and EV71-infected WT (n=9) and TLR7KO (n=8) mice were collected during recovery phase at day 12-15 (WT) or day 12-19 (KO) post-infection. Sera were heat-inactivated, serially diluted and incubated with EV71 virus (5000 TCID) followed by incubation with RD cells. The dilution titer completely preventing virus-induced cytopathic effects in RD cells is referred as serum neutralization titer. Data are shown as mean ± SD. **p < 0.01 by Mann-Whitney U test. N.D., not detected.

TLR7KO mice infected with EV71 showed a decrease of IgM and IgG in the spinal cord compared with WT mice as aforementioned. Analyzing the number of B cells (CD45^+^CD11b^-^B220^+^) in the spinal cord by FACS (**Figure 6A** and **Supplementary Figure 2**), we found that EV71-infected TLR7KO mice significantly reduced the frequency and the number of B cells in the spinal cord at day 7 and day 16 post-infection (**Figure 6A**), which might be associated with reduced levels of IgM and IgG in the spinal cord of EV71-infected TLR7KO mice as compared with EV71-infected WT mice (**Figure 5B**). TLR7 has been reported to play a role in the selection for high-affinity B cells in the germinal center reaction (30). Intriguingly, analysis of class-switched germinal center B (GC B) cells (CD45^+^B220^+^ IgM^-^IgD^-^CD95^+^CD38^int^) in the spleen revealed that GC B cells were significantly reduced in the TLR7KO mice during recovery phase as compared with WT mice (**Figure 6B** and **Supplementary Figure 2**). These results suggest that TLR7 deficiency affects the germinal center reaction and leads to decreased production of antigen-specific antibodies. The less levels of antibodies produced by TLR7KO mice should result in decreased neutralization of EV71. As expected, analyzing the titer of antibodies in serum to neutralize EV71 antigens, we found that antibodies from TLR7KO mice showed reduced ability on neutralization of EV71 as compared with WT mice (**Figure 7**).

### The Levels of FcγRs on Microglia Are Increased During EV71 Infection

**Figure 8:**
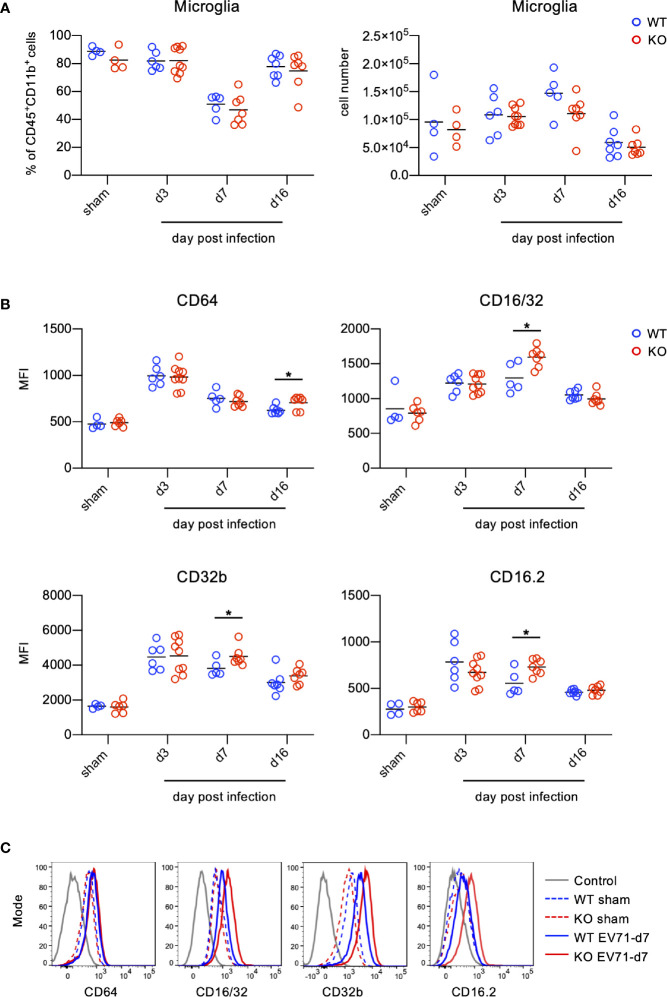
Microglia up-regulate FcγRs during EV71 infection. Spinal cord cells from EV71-infected mice were harvested, isolated and subjected to FACS analysis of FcγRs on microglia during EV71 infection. Microglia were identified as Ly6C^-^Ly6G^-^ cells within the myeloid cells (CD45^+^CD11b^+^). **(A)** The frequency of microglia within the myeloid cells (left panel) and the cell number of microglia (right panel) in the spinal cord of EV71-infected mice at different days post-infection are shown. **(B**, **C)** Microglia were subjected to the analysis of FcγR expression. Mean fluorescence intensity (MFI) of FcγRI (CD64), FcγRIII/II(CD16/32), FcγRIIb (CD32b) and FcγRIV (CD16.2) on microglia at different days post-infection is shown **(B)**. Representative histograms of FcγRI (CD64), FcγRIII/II(CD16/32), FcγRIIb (CD32b) and FcγRIV (CD16.2) expression on microglia at day 7 post-infection are shown **(C)**. Each symbol represents one mouse. Data shown are from 7 independent experiments. *p < 0.05 by Mann-Whitney U test.

Microglia are thought to be phagocytes in the CNS responsible for the removal of pathogens as well as cell debris. Antibodies not only neutralize pathogens, but also exert Fc-receptor-dependent antibody-mediated effector functions in phagocytes. Given that TLR7KO mice infected with EV71 show reduced IgG levels (**Figure 5B**) and that IgG likely binds to FcγRs on microglia during EV71 infection, we examined the levels of FcγRs on microglia in the spinal cord during EV71 infection. The frequency and the number of microglia between WT and TLR7KO mice were comparable in WT and TLR7KO mice infected with EV71 (**Figure 8A**). Measuring mean fluorescence intensity (MFI) of FcγRs on microglia by FACS, we found that the levels of FcγRs on microglia were increased in both TLR7KO mice and WT mice at day 3, day 7 and day 16 post-infection as compared with microglia of sham-infected mice (**Figures 8B, C**). Interestingly, the levels of FcγRIII (CD16/32), FcγRIV (CD16.2) and FcγIIB (CD32b) on microglia in TLR7KO mice were higher than those of WT mice at day 7 post-infection, while the expression level of FcγRI (CD64) in TLR7KO mice were higher than that of WT mice at day 16 post-infection (**Figures 8B, C**).

## Discussion

The outcome of the virus infection for infants and young children may become more vulnerable and unpredictable due to the concurrence of the infection and the immature immune responses. In this study, we investigated the role of TLR7 in EV71 infection in mouse pups (10-12 days old). It has been reported that TLR7 acts as a PRR for a variety of ssRNA viruses (15, 31–35) and that TLR7 regulates neuronal dendritic and axonal growth in the brain during development (36, 37). However, our study demonstrates that, despite that EV71 is an ssRNA virus, TLR7 is not the absolute PRR for the EV71 in the spinal cord and that EV71 mainly infected motor neurons in the spinal cord independent of TLR7. Interestingly, the most significantly different phenotype we observed is that TLR7KO mice showed delayed recovery from the limb paralysis as compared with WT mice. We further demonstrate that TLR7KO mice showed significantly reduced levels of IgM and IgG2 subtypes (IgG2a/c and IgG2b), important antibodies for anti-viral effect, in the spinal cord, and less ability to neutralize EV71 during their recovery from paralyzed limbs (> 14 days post-infection, which is equivalent to 4-week-old mice). The reduced production of IgG2 antibodies in EV71-infected TLR7KO mice weakens anti-viral humoral immunity to EV71, which is likely attributable, in part, to the delayed recovery compared with WT mice. It is plausible that TLR7 preferentially mediates anti-viral humoral immunity to EV71 in the spinal cord.

TLR7 does not seem to be the absolute PRR for EV71 in the spinal cord because both WT and TLR7 KO mice are able to induce most antiviral molecules and proinflammatory cytokines with similar levels at early stage of infection (day 3 post-infection), though the levels of some anti-viral molecules are lower in TLR7KO mice compared with WT mice (**Figure 3**). Some other PRRs recognizing RNA virus may act as PRRs for recognizing EV71 in motor neurons. Using a neural cell line for the study, Huang et al. recently showed that PRRs for EV71 are TLR3, TLR7 and MDA5 (38). Using animal study, our current study demonstrates that TLR7 is not the absolute PRRs for motor neurons. Additionally, our unpublished data showed that mice deficient in MAVS, a critical adaptor molecule for MDA5, showed comparable survival rate and disease severity as compared with WT mice, suggesting that MDA5 may not be the PRR for EV71. Whether TLR3 is the PRR for motor neurons remains to be validated by the animal study. Although TLR7 may not be the PRR for motor neurons, it may be an important PRR for other type of cells. For example, TLR7 has been reported as a PRR for macrophages recognizing EV71, in which HRS (hepatocyte growth factor-regulated tyrosine kinase substrate) is a key component for TLR7 signaling to induce inflammatory cytokines and anti-viral molecules by enhancing the TLR7 complex formation with TAB1 (39).

Infants and young children are particularly susceptible to EV71 infection, which is likely due to the immaturity of their immune responses. To mimic human infection by EV71, we infected mouse pups at age of 10-12 days old with EV71. Of note, mouse immune system is not fully mature until age of 3-4 weeks (28, 29). These IgG subtypes present at day 3 and day 5 post-infection (equivalent to the age of 14 and 16 days) in sera and in the spinal cords (**Figures 5D, E**) are supposed to be maternal antibodies, majorly IgG transferred from mother to children for passively protecting neonates and infants during the early time in life. This may be why we did not see the difference in antibody levels between EV71-infected WT and TLR7KO mice at earlier time points of the infection. We observed that mice were usually recovered from severe limb paralysis to clinical score equivalent to 1 (wobbling) at the age of more than 4-week-old, in which time the immune responses became mature. Therefore, IgG subtypes detected in the spinal cord during the recovery phase are considered generated from the adaptive immunity induced by EV71 infection. TLR7KO mice infected with EV71 showed significantly reduced IgG2a/c, IgG2b and IgM while increased IgG1 and IgG3 as compared with WT mice, suggesting that TLR7 play a critical role in humoral immunity to EV71 infection by generating Th1 associated antibodies- IgG2a/c and IgG2b, important for host defense to viral infection.

TLR7 has been reported expressed on B cells. Castiblanco et al. have shown that TLR7 signaling regulates selection in germinal center B cells by increasing somatic hypermutation and promotes germinal center B cells differentiating predominately into memory B cells rather than plasma cells (30). Clingan et al. also have demonstrated that TLR7KO mice show impaired germinal center reactions, resulting in a lesser extent to antibody responses during acute viral infection (40). In line with these notions, we observed that EV71-infected TLR7KO mice significantly reduced both frequency and number of class-switched germinal center B cells in the spleen during recovery phase (**Figure 6B**), contributing to the reduced levels of IgM and IgG in the spinal cord of TLR7KO mice (**Figure 5B**). Heer et al. have reported the critical role of TLR7 signaling in regulating anti-influenza B cell antibody isotype switching to IgG2 (41), and Miyauchi et al. have shown the importance of IgG2 responsible for protective immunity to lethal challenges with pathogenic H5N1 and pandemic H1N1 influenza virus strains (42). Our study also revealed the importance of TLR7 on the generation of IgG2 antibodies to host immunity to EV71 infection, as shown that IgG2 (IgG2a/c and IgG2b) levels in TLR7KO mice were significantly reduced compared with WT mice (**Figures 5B, C**). In addition, vaccine linked to a TLR7 ligand produces high levels of IgG2c, which protects mice from infection while antibodies of identical specificity, but of the IgG1, fail to do so (43). Consistent with this finding, we did observe that TLR7KO mice produced lower levels of IgG2c while higher levels of IgG1 (**Figures 5B, C**), leading to less protection of TLR7KO mice from EV71 infection, which may allow longer time for TLR7KO mice to recover from paralyzed limbs. Of note, it is important to have memory B cells in previously infected tissues since memory B cells will robustly produce high affinity antibodies upon re-infection (44). TLR7KO mice infected with EV71 indeed showed reduced B cells in the spinal cord (**Figure 6A**), which may impair the antibody production upon EV71 re-infection. Taken together, it is plausible that TLR7 plays a role in skewing B cells to generate IgG2a/c and IgG2b upon virus infection including EV71 infection. In the absence of TLR7, both number and functionality of B cells are affected, leading to delayed recovery from virus infection. In line with our finding on the critical role of TLR7 in antibody responses during viral infection, a clinical study showed that specific SNPs (single nucleotide polymorphisms) in TLR7 were associated with antibody responses to CMV glycoprotein B vaccine (45), further supporting the crucial role of TLR7 in anti-viral humoral immunity. Whether these SNPs of TLR7 impact humoral immune responses to EV71 warrants further study.

Virus-specific antibodies are critical for clearing the virus from CNS by neutralizing free virus and suppressing virus released from persistent infected neurons (46, 47). During acute viral infection of CNS, the major antigen-specific antibody subclass in the CNS are IgM and IgG, which are important for clearance of several neurotropic virus including West-Nile virus, Sindbis virus, and Rabies virus (47–49). In this study, we found that the reduced levels of IgG and IgM along with the reduced number of B cells in the spinal cord of TLR7KO mice may impair humoral immunity to EV71 infection, thus delaying recovery from EV71 infection. Chen’s group has shown that the CD4 T cell-independent antibody response promotes the survival of EV71-infected mice (50). Both their and our studies indicate the crucial role of antibodies in EV71 infection. If the residual virus in the CNS cannot be completely eradicated by antibodies in the early life, the residual virus may allow children to develop some neurologic sequelae in the later life. A study on a long-term follow-up of children infected with EV71 reveals that children infected with EV71 with CNS involvement show neurologic sequelae, delayed neurodevelopment and reduced cognition, further affecting their learning and behavior later (6). It is plausible that TLR7 signaling in regulating B cells to generate EV71 virus-specific antibodies, mainly IgG2 positioning in the CNS, are important for the clearance of residual virus or persistent RNA to suppress reactivation of virus, thus avoiding the complication leading to the neurological sequelae.

Microglia are considered phagocytes of the CNS and able to clear virus-infected cells and debris. The initiation of antibody-mediated effector function on phagocytes is governed by Fc portion of antibody *via* binding Fc portion to FcγRs on phagocytic cells. Similar to others’ report showing brain-resident microglia expressing FcγRs (51), we found that microglia in the spinal cord also express all four FcγRs including activating FcγRs (FcγRI, FcγRIII and FcγRIV) and inhibitory FcγR (FcγRIIB) (**Figure 8**). The engagement of immune complexes to FcγRs on microglia is supposed to induce FcγR activation to elicit effector functions, such as ADCC (antibody-dependent cellular cytotoxicity), ADCP (antibody-dependent cellular phagocytosis), to confer protective activity against pathogens (52–54). Mice have four subclasses of IgG, namely IgG1, IgG2a/c, IgG2b and IgG3. Mouse IgG2a/c has the strongest effector function followed by IgG2b while IgG1 and IgG3 have less potent effector function (55–57). IgG2a/c binds to all activating FcγRs with high affinity; IgG2b binds to FcγRIII and FcγRIV with high affinity while to FcγRI with low affinity (55, 56). IgG1 only binds to FcγRIII and IgG3 does not bind to any of FcγRs with significant affinity (55). IgG1 also binds to inhibitory FcγR with high affinity (58, 59). Of interest, TLR7KO mice infected with EV71 showed significantly increased levels of all FcγRs on microglia in the spinal cord compared with WT mice (**Figure 8**). Given that TLR7KO mice infected with EV71 showed less IgG2a/c and IgG2b (**Figures 5B, C**), we would expect that less IgG2a/c and IgG2b form less immune complexes, and then occupy less FcγRs and induce less FcγR aggregation for internalization, which leave more unoccupied FcγRs on cell surface and induce weak FcγR-mediated effector function. This is one explanation for the observation on reduced levels of FcγRs on microglia in the spinal cord of EV71-infected TLR7KO mice. Additionally, we found that microglia from EV71-infected TLR7KO mice produced less levels of TGF-β1 and ALOX15 (arachidonate 15-lipoxygenase) (**Supplementary Figure 3**), molecules important for resolving inflammatory and/or damaged tissues. Although EV71-infected WT and TLR7KO mice showed comparable numbers of microglia (**Figure 8A**), the functionality of microglia from EV71-infected TLR7KO mice seemed impaired as compared with EV71-infected WT mice (**Figure 8B** and **Supplementary Figure 3**). It is plausible that TLR7 on microglia in the spinal cord plays an important role in host defense to EV71 infection.

Our study shows that EV71 mainly infects lower motor neurons but not microglia and astrocytes in the spinal cord (**Figure 2**) and that TLR7 is important for regulating anti-viral humoral immunity (**Figures 5**–**7**). It is plausible that EV71-infected motor neurons become damaged/debris, which require being cleared and repaired by activated microglia. The Fc portion of anti-viral antibodies whose generation is regulated by TLR7 induces ADCP of microglia to exert the clearance and repair of damaged neurons or debris in the spinal cord. TLR7 in EV71 infection in the spinal cord does not seem to serve as a PRR for CNS cells, instead, TLR7 regulates the generation of anti-viral antibodies to activate microglia to exert their functionality during EV71 infection. Thus, our study demonstrates the beneficial effect of TLR7 on EV71 infection in the spinal cord. On the contrary, a recent study by Luo et al. has shown the detrimental effect of TLR7 on EV71 infection in the brain (18). The authors showed that EV71 preferentially infects astrocytes in the brain and induces neural pathogenesis *via* TLR7. The discrepancy in TLR7 function between the brain and the spinal cord during EV71 infection is currently unknown. It might be due to the difference in virus strain, mouse age, infection route, or properties of glia characteristics/function in different regions of the CNS and so on.

In sum, we have demonstrated that TLR7 is critical for regulating proper anti-viral humoral immunity to EV71 by promoting the production of IgG2a/c and IgG2b, which then bind to FcγRs on microglia to exert effector function. The critical role of TLR7 in humoral immunity against EV71 in the CNS warrants infants and young children infected with EV71 to eliminate the residual virus or viral RNA in the CNS, avoiding their developing CNS sequelae.

## Data Availability Statement

The datasets presented in this study can be found in online repositories. The names of the repository/repositories and accession number(s) can be found in the article/**Supplementary Material**.

## Ethics Statement

The animal study was reviewed and approved by the Institutional Animal Care and Utilization Committee at Academia Sinica.

## Author Contributions

Y-LL designed and performed experiments, analyzed data, and wrote the manuscript. M-YL, C-FC, YK, and H-EL performed experiments and analyzed data. F-AL analyzed proteomic data. J-RW provided crucial virus clone. Y-PH provided crucial animals. FL conceived and designed the study, supervised experiments, analyzed data, and wrote the manuscript. All authors contributed to the article and approved the submitted version.

## Funding

This study was supported by grants from the National Science Council in Taiwan (NSC99-2321-B-001-035, NSC100-2321-B-001-025, NSC101-2321-B-001-013).

## Conflict of Interest

The authors declare that the research was conducted in the absence of any commercial or financial relationships that could be construed as a potential conflict of interest.
