# Supplementary material for: TLR7 Is Critical for Anti-Viral Humoral Immunity to EV71 Infection in the Spinal Cord
Source: Front Immunol. 2021 Feb 18;11:614743. doi: 10.3389/fimmu.2020.614743 (PMC7935532; doi:10.3389/fimmu.2020.614743)
Supplement: Supplementary file 1 [file DataSheet_1.pdf]

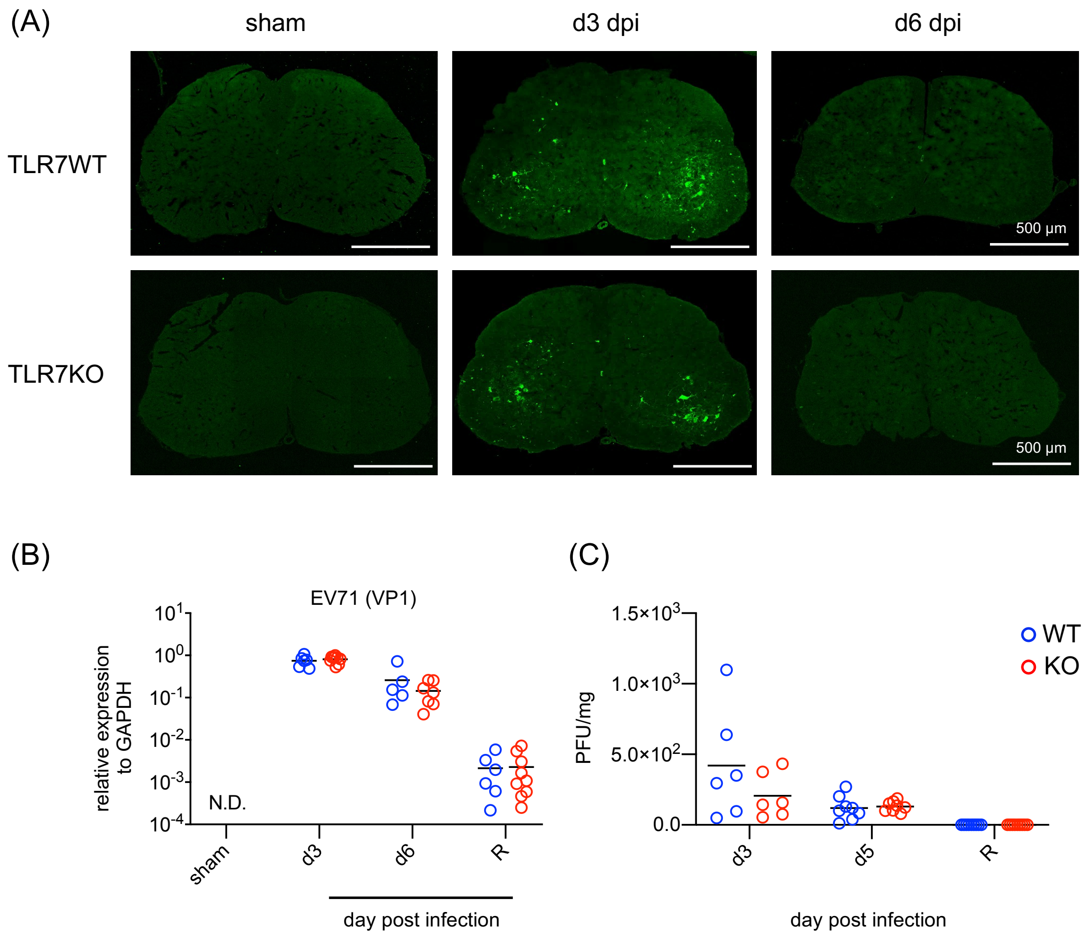

Supplementary Figure 1. WT and TLR7KO mice infected with EV71 show similar virus load in the spinal cord.

(A) Cryosections of the lumbar spinal cord from sham-infected and EV71-infected WT and TLR7KO mice were subjected to antigen retrieval followed by sequentially staining with rabbit anti-VP1 antibody and Alexa Fluor488-conjugated goat anti-rabbit (green) (dpi, day post infection). (B) EV71-infected WT and TLR7KO mice were sacrificed on different days post infection. Spinal cords were collected and subjected to total RNA isolation followed by first-strand cDNA synthesis. Virus load was analyzed by VP1 expression of EV71 using quantitative PCR. (C) Virus load in the spinal cord of EV71-infected WT and TLR7KO mice was determined by plaque assay. Each symbol represents one mouse. N.D., not detected. R, recovery phase (d9-d18 post infection).

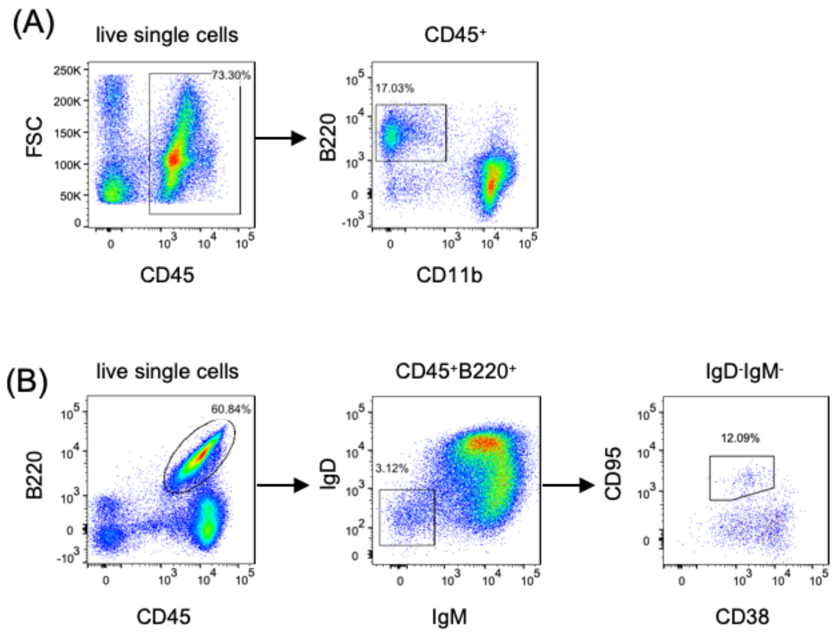

Supplementary Figure 2. Gating strategies for identifying B cells in spinal cords and class-switched germinal center B cells in spleens.

Spinal cord cells and splenocytes from EV71-infected mice were isolated and subjected to FACS analysis of B cells and B cell subsets. Spinal cord B cells were identified as $CD45^{+}B220^{+}CD11b^{-}$ cells (A). Class-switched germinal center B cells in spleens were identified as $CD45^{+}B220^{+}IgD^{-}IgM^{-}CD95^{+}CD38^{int}$ cells (B).

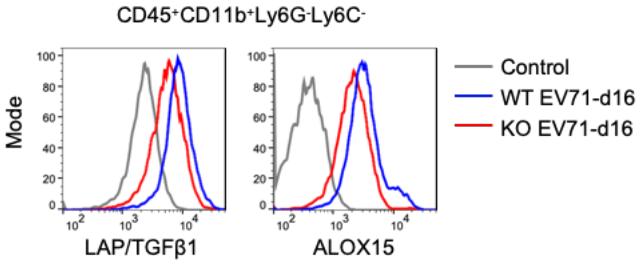

Supplementary Figure 3. TLR7-deficient microglia produce less levels of TGF-β1 and ALOX15 in the spinal cord.

EV71-infected WT and TLR7KO mice were sacrificed at day 16 post-infection. Spinal cord cells were isolated and subjected to surface staining of microglia markers ($CD45^{+}CD11b^{+}Ly6G^{-}Ly6C^{-}$) followed by intracellular staining of TGF-β1 and ALOX15. Representative histograms are shown.

Supplementary Table 1. Primer pairs used for qPCR.

Gene	Forward	Reverse
IRF7	TCCAGCGAGTGCTGTTTGGA	CGAGCCTCGTTCAGCCA
IFIT1	AAACCCAGAGAACAGCTACCA	GCATCCCCAATGGGTTCTTG
OAS1a	CCCTATCTGACACATTAGCGGT	ATATCTATGGTCCCCCAGCCT
IL-12a	CGGTCCAGCATGTGTCAATCA	CACCATGTCATCTGTGGTCTTC
IFNα	ARSYTGTSTGATGCARCAGGT	GGWACACAGTGATCCTGTGG
IFNβ	AGGGCGGACTTCAAGATC	CTCATTCCACCCAGTGCT
Mx1	CCAACTGGAATCCTCCTGGAAAA	CCTTCTCCTCATAGTGCCTGC
ISG15	ACTCCATGACGGTGTCAGAAC	TTCGTTCCTCACCAGGATGC
OAS1b	GCCGAATGAGGGCCTCTAAA	AAGCCCCATAAAGCAGATCG
TNF-α	TTCTCATTCCTGCTTGTGGCA	TGATGAGAGGGAGGCCATTTG
IL-1β	TGTAATGAAAGACGGCACACC	TCTTCTTTGGGTATTGCTTGG
CXCL10	TGAGCAGAGATGTCTGAATC	TCGCACCTCCACATAGCTTACAG
EV71	GTGGCAGATGTGATTGAGAG	GTTATGTCTATGTCCCAGTT
GAPDH	TACTTGGCAGGTTTCTCCAG	GTCGTGGAGTCTACTGGTGT

Supplementary Table 2. Antibodies used in immunofluorescence staining and staining procedures.

Steps	Primary antibody	Brand	Secondary	Brand
1	Goat anti-choline acetyl transferase	Millipore	Alexa Fluor 594 Donkey anti-goat	Molecular Probes
2	Mouse anti-NeuN	Millipore	Alexa Fluor 488 Goat anti-mouse	Molecular Probes
3	Image capture			
4	Antigen retrieval			
5	Rabbit anti-EV71 VP1	GeneTex	Cy3 Goat anti-rabbit	Molecular Probes
6	Image capture and processing			

Supplementary Table 3. Fluorochrome-conjugated antibodies and the viability dye used in flow cytometry assay.

Target species	Antigen	Clone	Fluorochrome	Brand
mouse	CD16/32	2.4G2	none	BD Pharmingen
mouse/human/rat	NeuN	EPR12763	Alexa Fluor 488	Abcam
mouse	ACSA2	REA969	FITC	Miltenyi
mouse/human/rat	O4	REA576	APC	Miltenyi
mouse	CD90.1(Thy1.2)	53-2.1	Biotin	BD Pharmingen
mouse	CD64	X54-5/7.1	Brilliant Violet 605	BioLegend
mouse	CD16/32	93	eFluor450	eBioscience
mouse	CD32b	AT130-2	APC	eBioscience
mouse	CD16.2	9E9	PerCP/Cyanine5.5	BioLegend
mouse	CD45	30-F11	Brilliant Violet 785	BioLegend
mouse/human	CD11b	M1/70	FITC, PE	BioLegend
mouse	Ly6C	HK1.4	eFluor450, Brilliant Violet 510	eBioscience, BioLegend
mouse	Ly6G	1A8	Brilliant Violet 650	BioLegend
mouse/human	B220	RA3-6B2	BUV395	BD Pharmingen
mouse	IgD	11-26c.2a	FITC	BioLegend
mouse	IgM	II/41	PE-Cyanine7	eBioscience
mouse	CD95	15A7	PerCP-eFluor710	eBioscience
mouse	CD38	90/CD38	PE	BD Pharmingen
mouse	TLR7	A94B10	PE	BD Pharmingen
mosue	LAP	TW7-16B4	PerCP-eFluor710	eBioscience
human, mouse, rat	Alox15	rabbit polyclonal	Alexa Fluor647	Bioss
Streptavidin			BUV496	BD Horizon
Fixable viability Dye			eFluor780	eBioscience

Supplementary Table 4. A list of proteins with abundance ratio (KO/WT) <0.75 fold during recovery phase.

UniProt Accession	Gene Name	Abundance Ratio (KO/WT)
F6TQW2	immunoglobulin heavy constant gamma 2C (Ighg2c)	0.344
A0A0G2JFE9	immunoglobulin heavy variable 1-76 (Ighv1-76)	0.566
A0A0B4J1J5	immunoglobulin heavy variable V9-3 (Ighv9-3)	0.604
P01837	immunoglobulin kappa constant (Igkc)	0.608
A0A075B6A0	immunoglobulin heavy constant mu (Ighm)	0.628
A0A0G2JE47	immunoglobulin kappa variable 8-28 (Igkv8-28)	0.65
A0A075B5V1	immunoglobulin heavy variable 1-31 (Ighv1-31)	0.657
Q9ESX4	zinc finger, CCHC domain containing 17 (Zcchc17)	0.673
P24526	peripheral myelin protein 2 (Pmp2)	0.696
Q5SVL6	RAP1 GTPase activating protein 2 (Rap1gap2)	0.709
P01644	immunoglobulin light chain variable region (Ce9)	0.719
Q62093	serine/arginine-rich splicing factor 2 (Srsf2)	0.72
A0A0A6YWX0	immunoglobulin heavy variable V1-19 (Ighv1-19)	0.728
E9Q7N4	diacylglycerol kinase kappa (Dgkk)	0.733
Q9R020	zinc finger, RAN-binding domain containing 2 (Zranb2)	0.733
G3UY45	lysine (K)-specific methyltransferase 2C (Kmt2c)	0.738
A0A0R4J1E3	drebrin 1 (Dbn1)	0.742
E9Q7D5	Rho guanine nucleotide exchange factor (GEF) 5 (Arhgef5)	0.743
P47713	phospholipase A2, group IVA (cytosolic, calcium-dependent) (Pla2g4a)	0.747
Q8VE52	opioid growth factor receptor-like 1 (Ogfrl1)	0.748
